# Leaving behind the Mucosa: Advances and Future Directions of Intestinal Ultrasound in Ulcerative Colitis

**DOI:** 10.3390/jcm12247569

**Published:** 2023-12-08

**Authors:** Alberto Barchi, Arianna Dal Buono, Ferdinando D’Amico, Federica Furfaro, Alessandra Zilli, Gionata Fiorino, Tommaso Lorenzo Parigi, Laurent Peyrin-Biroulet, Silvio Danese, Mariangela Allocca

**Affiliations:** 1 Gastroenterology and Endoscopy, IRCCS Ospedale San Raffaele, Vita-Salute San Raffaele University, 20132 Milan, Italy; barchi.alberto@hsr.it (A.B.); damico.ferdinando@hsr.it (F.D.); furfaro.federica@hsr.it (F.F.); zilli.alessandra@hsr.it (A.Z.); fiorino.gionata@hsr.it (G.F.); daneses@hotmail.com (S.D.); 2 IBD Center, Humanitas Research Hospital-IRCCS, Via Manzoni 56, Rozzano, 20089 Milan, Italy; arianna.dalbuono@humanitas.it; 3 Department of Biomedical Sciences, Humanitas University, Pieve Emanuele, 20072 Milan, Italy; 4 Department of Gastroenterology and Inserm NGERE U1256, University Hospital of Nancy, University of Lorraine, 54500 Vandoeuvre-lès-Nancy, France; peyrinbiroulet@gmail.com; 5 Department of Gastroenterology, Nancy University Hospital, 54500 Vandœuvre-lès-Nancy, France; 6 INFINY Institute, Nancy University Hospital, 54500 Vandœuvre-lès-Nancy, France; 7 Federation Hospitalo-Univeristaire-CURE, Nancy University Hospital, 54500 Vandœuvre-lès-Nancy, France; 8 Groupe Hospitalier Privé Ambroise Paré-Hartmann, Paris IBD Center, 92200 Neuilly-sur-Seine, France; 9 Division of Gastroenterology and Hepatology, McGill University Health Centre, Montreal, QC H4A 3J1, Canada

**Keywords:** intestinal ultrasound, ulcerative colitis, IBD monitoring, bowel thickness

## Abstract

Inflammatory Bowel Diseases (IBD), mainly Ulcerative Colitis (UC) and Crohn’s Disease (CD), are disorders characterized by chronic inflammation with severe morbidity and long-term disabling quality of life outcomes. UC mainly affects the mucosal and sub-mucosal layers of the colon, without embracing the peri-intestinal structures. Considering the predominant mucosal location of UC inflammation, the implementation of transmural evaluation by cross-sectional imaging techniques, mainly Intestinal Ultrasound (IUS), has been left behind for ages, especially if compared to CD. Nevertheless, studies analyzing intestinal ultrasound parameters accuracy in disease activity detection reported a good-to-optimal correlation of IUS markers with colonic inflammation, suggesting comparable feasibility of IUS monitoring in UC as in CD. The easy-to-use, costless and point-of-care available status of IUS is therefore crucial in order to improve the diagnostic process and, according to the recent literature, to monitor the response to treatment leading to speeding up decision making and therapy adjustments. Recent studies have demonstrated the correlation between transmural healing in UC with favorable outcomes even in the long term. An evidence gap still exists in the assessment of the rectum, with trans-perineal ultrasound (TPUS) a potential answer to reach a more precise evaluation of rectal inflammation. Eventually, IUS is also increasingly showing promises in emergent or post-surgical UC settings, considering various efforts put in line to demonstrate its feasibility in predicting response to salvage therapy for surgery avoidance and in studying inflammation relapse after procto-colectomy with ileo-pouch–anal anastomosis (IPAA) creation.

## 1. Introduction

Ulcerative colitis (UC) is a chronic inflammatory bowel disease (IBD), mainly involving the superficial mucosal layer of continuous colonic segments, including the rectum [1]. UC incidence and prevalence are constantly increasing, suggesting an overall improvement in the diagnostic process, concomitantly to long-lasting survival [1]. The Selecting Therapeutic Targets in Inflammatory Bowel Disease (STRIDE) consensus established the treat-to-target strategy with tight monitoring of the UC course, possibly adjusting therapies to stop disease progression [2]. The recently adjourned STRIDE II initiative has implemented the importance of non-invasive monitoring in UC control through periodical fecal and serum biomarkers evaluation and using intestinal ultrasound (IUS) assessment [3]. IUS has demonstrated feasibility in IBD diagnosing and monitoring, particularly in Crohn’s disease (CD), thanks to high patient tolerability and elevated standard of accuracy [4]. The nature of UC is considered mainly a “mucosal” disease, involving predominantly the lower segments of the colon, easily reachable with sigmoidoscopy, and has obscured its use for years. Recently, the use of IUS in UC has been revolutionized, provided that bowel wall thickness (BWT) has been evaluated as the most relevant marker of inflammatory activity in IBD, even in UC [5]. Together with the other ultrasound features compatible with gut inflammation, the adoption of IUS in UC both in routine practice and in clinical trials has grown enormously [6]. IUS in UC has gained rapid consensus among IBD experts for its good correlation with endoscopic activity and for accurately reflecting the stage of UC inflammation [7]. In addition, the “point-of-care” availability of this technique allows prompt therapeutic adjustments, improving clinical decision making and patient outcomes. For this reason, recent guidelines state the IUS’s role in detecting activity and monitoring treatment response in UC, even if the concept of transmural remission is not yet standardized, as in CD [8,9]. This review aims to present all the latest insights into the use of IUS for UC assessment, including activity detection and evaluation, and the tight monitoring of treatment response.

### Review Criteria

Pubmed, Embase, and Scopus databases were screened up to 30 September 2023 in order to identify relevant studies exploring the accuracy, sensitivity, specificity and overall feasibility of ultrasound techniques in the management and follow-up of UC. The following search terms were used: ‘ultrasound’, ‘bowel ultrasound’, ‘intestinal ultrasound’ and ‘standard intestinal ultrasound’, combined with ‘UC’, ‘ulcerative colitis’, ‘colitis’, ‘IBD’, ‘post-surgical UC’, ‘pouch’ and ‘IPAA’. Only articles published in English were considered. In addition, references to original articles and relevant reviews were screened to find additional publications. No publication date restrictions were applied. We excluded case reports, case series and any irrelevant abstract. From the literature search, 1534 articles were extracted and only 67 were included.

## 2. IUS in UC Activity Assessment

### 2.1. Colonic Evaluation

**Figure 1:**
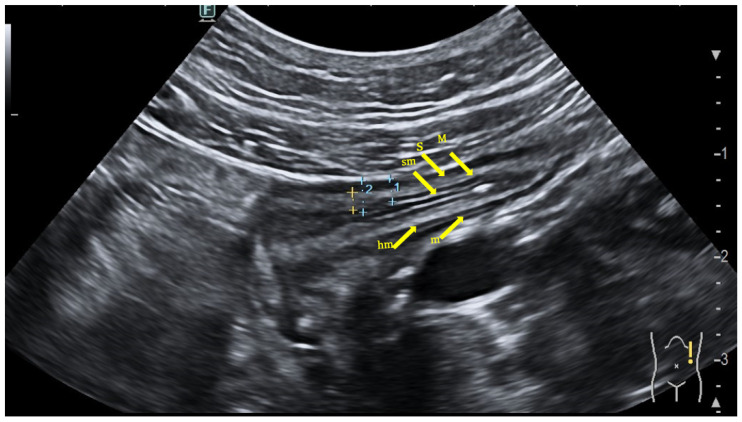
Bowel wall stratification at bowel ultrasound evaluation. hm: hyperechoic lumen interface of the mucosa; m: mucosa (hypoechoic); sm: submucosa (hyperechoic); M: muscolaris propria (hypoechoic); S: serosa (hyperechoic).

**Figure 2:**
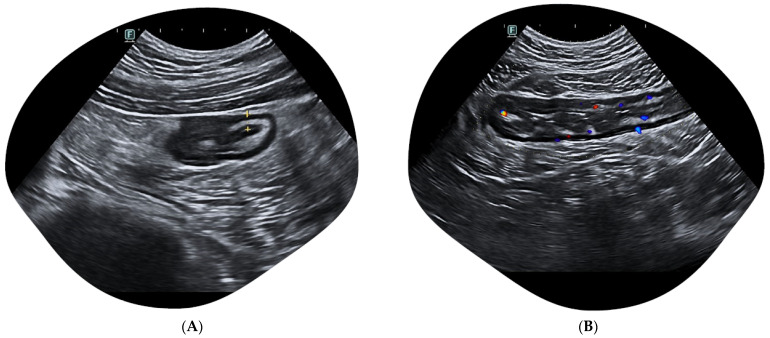
Ultrasound presentation of an active left-sided ulcerative colitis. (**A**) Increased BWT (3.4 mm) in a mild active left-sided ulcerative colitis, in the descending colon. (**B**) Severe acute ulcerative pancolitis: the sigmoid colon results severely inflamed with a CDS 3 according to Limberg score, complete disruption of BWS (hyperechoic spots in the mucosa/submucosal interface suggesting deep ulcerations) and increased BWT.

First reports on the feasibility of IUS parameters in UC evaluation dated back to the 1980s, contemporary to CD studies [10]. Interestingly, Limberg and colleagues described a sensitivity in activity detection of IUS in UC patients around 89% in 1989 [11]. The comprehensive evaluation of UC activity, similarly to CD, relies on the correct step-by-step performance of the whole procedure: generally, it is advisable to start the examination by providing a broad picture of the whole abdomen with a convex low-frequency probe (5–6 MhZ) to spot target lesions, and completed with a microconvex or linear high-frequency probe (11–14 MhZ) usually starting from the sigmoid colon in the left iliac fossa [12]. Fasting is not necessary, even if advisable being able to reduce luminal content, and decrease peristalsis [13]. The operator has to be able to distinguish the five concentric layers of bowel wall by their echogenicity (Figure 1): the hyperechoic mucosa is the most inner layer, followed by hypoechoic submucosal, the hyperechoic muscular layer and the echogenic interface between the serosa with the anatomic surroundings as the most outer layer (Figure 1) [13]. The main parameter to be addressed in IUS is Bowel Wall Thickness (BWT), measured in all colonic segments, reflecting the grade of mucosal inflammation [14]. Together with BWT Color Doppler Signal (CDS) is also an accurate parameter of intestinal inflammation degree. Vascular signals are usually graded using the Limberg score [15]. It consists of a semi-quantitative score where 0 points stands for a normal thickened wall with the absence of vascular spots, 1 point for a thickened wall without vascularization, 2 points for small spots of CDS, 3 points for larger vascular signs still within the bowel layers, while 4 points are given for long stretches of CDS extending to the surrounding tissues [15]. Bowel Wall Stratification (BWS), a parameter describing the loss of interface between the various bowel layers, represents a direct sign of mucosal damage reflecting the presence of erosions or even deep ulcers, disrupting the echo pattern [14]. Indirect signs seemingly relevant to be evaluated in order to spot mild inflammatory activity are swollen lymph nodes or peri-intestinal mesenteric fat (i-fat) (Figure 2) [14]. The most recent systematic review evidencing the use of IUS in UC patients identified 50 studies reporting IUS features and clinical characteristics [16]. One of the systematically reviewed studies by Parente et al. dating back to 2003 resulted to be the largest prospective UC cohort (84 patients) assessed until then, reporting BWT as the main ultrasound marker of disease activity, evidencing an overall sensitivity of 87% in detecting inflammation in the descending/sigmoid colon, using colonoscopy as the reference standard [17]. More recently a Japanese prospective multicenter study, enrolling 154 UC patients with colonoscopy as the reference standard, found a moderate correlation between IUS findings (mainly BWT, wall stratification and presence of ulcerations) and colonoscopy findings expressed as a Kappa (K) statistic of 0.55 (*p* < 0.001) [18]. The authors also provided the correlation between IUS findings and both clinical activity expressed with the clinical activity index (CAI) (*r* = 0.40, *p* < 0.001) and histology grade (*r* = 0.35, *p* < 0.001) [18]. In addition to the large sample size and the prospective design, this study used unstandardized clinical and endoscopic scores as reference comparisons. A more recent multicenter cross-sectional study prospectively enrolling 60 UC patients with overall 207 evaluated colonic segments, was able to identify a BWT cut-off value of 3.2 mm in predicting moderate-to-severe endoscopic activity (expressed as Endoscopic Mayo score [EMS] of 2–3) with an Area Under Curve (AUC) of 0.946 [19]. The authors furthermore reported CDS and loss of colonic haustration as surrogates of disease activity (EMS 2–3). Moreover, the inter- and intra-class correlation (ICC) between operators resulted substantial both for BWT than CDS (ICC >0.8, *p* < 0.001 and K > 0.60, *p* < 0.001, respectively) [19]. Similar consistent results were obtained by Allocca and colleagues, in a cohort of 53 prospectively enrolled UC patients, reporting BWT (*p* < 0.0001), CDS (*p* < 0.0001), enlarged mesenteric lymph nodes (*p* < 0.04) and hypoechoic pericolic fat (*p* < 0.01), as well as correlated with endoscopic activity (Mayo score > 2). The authors additionally identified BWT (1 mm increase, odds ratio [OR]: 4.05, *p* = 0.01) and CDS (OR: 7.99, *p* = 0.09) as predictors of endoscopic inflammation grade at multivariable analysis [20]. Moreover, the inter-observer agreement between operators for IUS features reached 86% [20]. The efficiency of IUS has also often been analyzed in combination with other non-invasive parameters of disease activity, such as blood biomarkers. Antonelli and colleagues evaluated 51 active UC patients reporting a good correlation between IUS parameters (mainly BWT) with C-reactive protein (CRP) levels (*p* < 0.0001). Other studies comparing CRP and IUS findings reported similar results [21,22]. The main studies describing ultrasound evaluation in UC activity assessment are displayed in Table 1. The seemingly high concordance between IUS findings and endoscopic appearance led to the recent design of prospective studies with the aim of developing IUS activity scores to standardize the assessment of UC features, as already implemented in CD [23]. First attempts in a codification of UC ultrasound parameters have been exploited in the past but with a lack of methodological rigor. Initial prospective studies built scores based only on bowel thickness measures, clustering patients according to BWT values [24,25] or on disease extent [26]. More recently, Allocca and colleagues developed the first, rigorous activity score for UC, the “Milan” Ultrasound criteria (MUC) [20]. MUC was defined as 1.4 × BWT (mm) + 2 × CDS, where CDS = 1 if present, and CDS = 0 if absent. In the same study at Receiving Operator Characteristic (ROC) analysis, a MUC > 6.2 was established as the optimal cut-off for disease activity [20]. Thanks to these findings the most recent guidelines on non-invasive assessment in IBD suggest IUS as a reliable tool in disease activity assessment, especially in left-sided pancolitis [9]. More large prospective studies or Randomized Controlled Trials (RCT) must be designed to implement an efficient strategy and methodology to implement IUS assessment in the diagnostic process of UC. A relevant step toward this goal has been accomplished by the International Bowel Ultrasound (IBUS) group, which has developed a core curriculum for the training in IUS performance with standardized targets and learning goals (IBUS; www.ibus-group.org). Another improvement could be represented by the development of easy-to-perform and fast techniques involving ultrasound evaluation for UC. Recently, Rispo et al. reported the diagnostic and monitoring accuracy of a hand-held IUS (HHIUS) in 86 UC patients, prospectively enrolled, finding comparable inter-observer agreement on MUC evaluation between standard IUS and HHIUS with a K Coehn test of 0.86, and no statistically significant difference in BWT outcomes between the two techniques [27].

### 2.2. The Challenge of Rectum Evaluation

**Figure 3:**
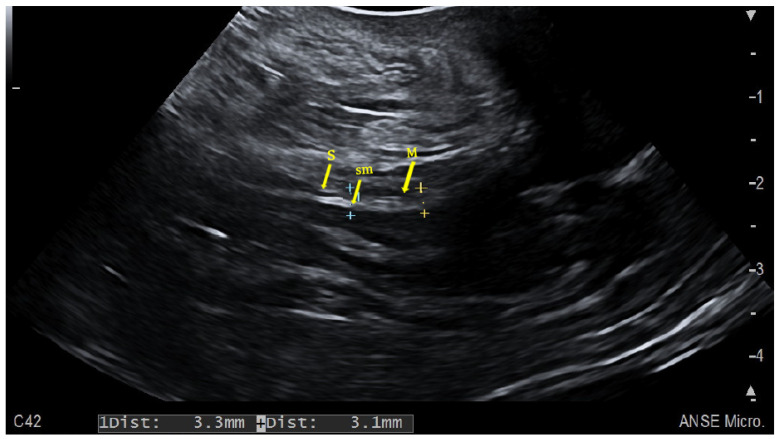
Trans-perineal Ultrasound (TPUS) window in a mild active ulcerative proctitis. sm: submucosa (hyperechoic); M: muscolaris propria (hypoechoic); S: serosa (hyperechoic).

If colonic evaluation has gained rapid consensus among IBD experts in UC extent and severity evaluation, the assessment of the rectum still represents a critical challenge when performing IUS [28]. Being the most involved gut tract in UC, the difficulty in identifying and correctly assessing the inflammation grade represents a real diagnostic gap [28], and it is prominent due to the disturbing interface with several organs (the bladder in particular) and its profound location in the pelvis [17]. In the most recent systematic review on IUS in UC [16], the authors described a low accuracy of IUS in detecting rectum activity. Parente et al. reported an 80% failure rate in the detection of rectal activity in UC patients who underwent colonoscopy and IUS concomitant evaluation [17]. More recently, a comprehensive metanalysis by Sagami et al. [29] evidenced a diagnostic OR of increased BWT in disease activity detection, significantly decreasing from the right colon (86.4) to the rectum (6.6) [29]. The pooled sensitivity for IUS in rectal assessment resulted considerably inferior to all other colonic segments (74.5% versus 86.4%) [29]. For ages, the main methodology used in disease extent evaluation in UC, especially for rectal activity, has been double-contrast X-ray enema and leucocyte-labeled scintigraphy, showing discrete levels of accuracy (up to 90%) [22]. The high costs and radioactive exposure have always limited these techniques in UC [26]. Therefore, the urgent need for non-invasive, accurate cross-sectional imaging methods for rectal activity assessment is pending [30]. Trans-perineal Ultrasound (TPUS) is a novel technique recently spreading in the IBD field [12]. Its use is proven efficient in CD, especially in complex settings like peri-anal disease, abscesses and fistula detection [31]. The patient is bent on left lateral decubitus with the linear probe leaned on the perineum in the peri-anal region. The ultrasound evaluation investigates the rectum, both external and internal anal sphincter, the pubis symphysis, the bladder, the prostate or the vagina [12]. Coherently with ultrasound parameters in the trans-abdominal examination, TPUS, BWT and CDS are also the main markers to be assessed (Figure 3). Sagami and colleagues designed a pilot study of TPUS in ulcerative proctitis patients to determine accuracy in activity detection [32]. A total of 51 patients underwent TPUS examination together with IUS and colonoscopy as the reference standard. The correlation between BWT and CDS with EMS in the rectum resulted in consistently higher TPUS (ρ BWT = 0.7204, *p* < 0.0001; ρ CDS = 0.6619, *p* < 0.0001) compared to trans-abdominal IUS (ρ BWT = 0.2989, *p* = 0.0314), or ρ CDS = 0.0649, *p* = 0.6442) [32]. Concerning accuracy, BWT ≥ 4 mm in the rectum at TPUS showed a sensitivity of 100%, a specificity of 45.8% with an AUC of 90.4% versus a sensitivity of 96.3%, a specificity of 12.5% with an AUC = 0.667 at trans-abdominal IUS [32]. The comparison between TPUS and trans-abdominal IUS was statistically significant (*p* < 0.0007). The authors interestingly examined the correlation between TPUS parameters and histological activity (defined as Geboes score > 2.1, Robarts histopathological index > 6, the Nancy score > 1), finding sensitivity up to 96% with AUC of 89% for a BWT > 4 mm. These outcomes could potentially represent a turning point in implementing TPUS in histological healing assessment [32]. Recently, in the field of pediatric UC, Tokushima and colleagues compared TPUS and colonoscopy in rectal activity assessment in 87 UC patients and 44 non-IBD proctitis patients. The multivariate analysis identified a novel metric called “microvascular signal at wall circumference” (MSWC) (OR 119.3; 95% CI, 10.21–5646, *p* = 0.002), together with BWT (OR, 8.80; 95% CI, 2.10–75.3, *p* = 0.01), as predictors of endoscopic activity [33]. BWT ≥ 4.5 mm was found to spot rectal activity in UC with a sensitivity of 84.2% and a specificity of 93.3% [33]. Future studies are warranted to explore the feasibility of TPUS in rectal assessment with UC.

**Table 1:** Studies assessing IUS performance in disease activity detection in UC.

Study	Study Type	Cohort N.	Clinical Context	Reference Standard	Ultrasound Score	Major Findings	Reference
Allocca et al. (JCC 2018)	Prospective single center	53	Active and inactive UC	CS	MUC: 1.4 × BWT (mm) + 2 × CDS	✓ Median BWT 3.0 mm vs. 5.0 mm between EMS 0–1 and EMS 2–3 (*p* < 0.0001) ✓ Also CDS (*p* < 0.0001), BWS (*p* < 0.01) and ↑ lymph nodes (*p* < 0.04) between EMS 0–1 and EMS 2–3 ✓ BWT (per 1 mm ↑, OR 4.05, 95% CI 1.37–11.9; *p* = 0.01) and CDS (OR 7.99, 95% CI 0.67–94.4; *p* = 0.09) ✓ Se and Sp of BWT > 3 mm were 89% and 87%	[20]
Kinoshita et al. (J Gastroenterol 2019)	Prospective multicenter	156	Active and inactive UC	CS	NA	✓ IUS could visualize colonic segments better than CS (*p* < 0.001) ✓ IUS and CS significant concordance for maximum grades (weighted *k* = 0.47, *p* < 0.001) and for all segments (weighted *k* = 0.55, *p* < 0.001)	[18]
Bots et al. (JCC 2021)	Prospective single center	60	Active and inactive UC	CS	UC-IUS index: [BWT > 2 mm, 3 mm and 4 mm (1-2-3 pts)] + [CDS spots (1 pts), stretches (2 pts)] + (abnormal haustration 1 pts) + (i-fat 1 pts)	✓ BWT = 2.1 mm cut-off with Se 82.6%, Sp 93%, AUC 91% to distinguish EMS0 and EMS1-3; BWT = 3.2 mm cut-off woth Se 89.1%, Sp 92.3%, AUC 94.6% to distinguish EMS 0–1 and EMS 2–3 ✓ ICC 0.917, 95% CI 0.853-0.948, *p* < 0.001 for BWT measurements ✓ CDS ≠ 0 predicted EMS ≠ 0 with OR 14, 95% CI 6.8–28.7, *p* < 0.001 ✓ Loss of haustration and i-fat predicted EMS ≠ 0 with OR 126.2, 95% CI 36.3–438.7, *p* < 0.001 and OR 34, 95% CI 6.0–191.8, *p* < 0.001, respectively	[19]
Sagami et al. (CGH 2021)	Metanalysis	1101	Active and inactive UC	CS	NA	✓ Overall segments BWT Se and Sp 86.4% and 88.3%. Diagnostic OR for right, transverse, left colon and rectum OR (95% CI): 86.4 (19.8–376.8), 60.0 (13.9–259.1), 59.5 (14.0–252.5), 6.6 (1.4–32.1), respectively ✓ Very high NPV of BWT (92.7%) ✓ Pooled Se and Sp in rectum 74.5% and 69.5% ✓ CDS and BWS true positive OR 7.39 (95% CI, 4.73–11.54), *p* < 0.0001 and 55.18 (95% CI, 32.48–93.76), *p* < 0.0001	[29]

AUC: Area Under Curve; BWS: Bowel Wall Stratification; BWT: Bowel Wall Thickness; CI: Confidence Intervals; CDS: Color Doppler Signal; CS: Colonoscopy; EMS: Endoscopic Mayo Score; ICC: Inter-observer agreement; i-Fat: Fat Wrapping; IUS: Intestinal Ultrasound; MUC: Milan Ultrasound Criteria; NPV: Negative Predictive Value; OR: Odds Ratio; pts: points; Se: Sensitivity; Sp: Specificity; UC: Ulcerative Colitis.

## 3. Bowel Ultrasound in UC Monitoring and Therapeutic Response Assessment

### 3.1. Tight Monitoring in UC: Preventing Colorectal Cancer (CRC) and Surgery Sparing 

Similarly to CD, the efficient monitoring of UC activity over time in a fast, easy-to-perform, point-of-care manner is crucial, given the high risk of cancer development or surgical risk of UC [1]. IUS provided as a “point-of-care” technique [34], potentially available at every clinical outpatient visit, performed by a trained IBD ultrasound specialist, could increase significantly the rapidity of therapeutic adjustments [35]. For this reason, the most recently updated STRIDE II initiative has upgraded IUS as a non-invasive tool critically useful in UC monitoring, even if not reaching a clear consensus on timing and positioning in the “treat-to-target” strategy due to lack of prospective data [3]. In fact, the concept of “transmural healing” in UC was classified as the last goal in order of relevance in UC, due to the prominent mucosal nature of this disease compared to CD [3]. To date, few studies have been carried out to demonstrate IUS feasibility in tight monitoring of UC, especially if compared with a reference standard of colonoscopy [22,36,37]. The validation study of MUC criteria for disease activity assessment by Allocca and colleagues evaluated 98 UC patients prospectively enrolled and underwent IUS evaluation and colonoscopy. The authors first confirmed a significant correlation between MUC and endoscopic Mayo subscore (ρ = 0.65; *p* < 0.001). Additionally, using Cox regression analysis, the MUC score resulted in predictions of negative outcomes of the disease over time (Hazard Ratio (HR) 3.87, *p* < 0.001), registering 98% of patients with MUC > 6.2 showing a negative course of disease versus only 48% with MUC < 6.2 (*p* < 0.001) [38]. MUC > 6.2 optimally predicted the need for corticosteroid therapy (HR: 7.20, *p* = 0.066) and the need for surgical treatment (*p* = 0.019) [38]. This was the first study evidencing a potential correlation between IUS parameters and negative outcomes of UC long term, suggesting a clear role of IUS as a fast and efficient assessment tool in UC monitoring [39]. Recently, Piazza and colleagues prospectively followed up with 141 patients for a median of 21.9 months, reporting a higher MUC value in those patients undergoing colectomy for persisting refractory disease versus non-operated patients (9.4, 8.9–11.1 versus 6.4, 4.2–8.9, *p* ≤ 0.001), together with mean BWT values (5.3, 4.9–6.7 mm versus 4.1, 3.0–5.0 mm, *p* ≤ 0.001) [40]. Moreover, no MUC ≤ 6.2 patients at baseline later underwent surgery compared to 16.4% of patients showing MUC ≥ 6.2 (*p* < 0.001) [40]. Notably, MUC, over EMS and even BWT, was the only significant predictor of Cox proportional hazard regression analysis (1.48, 95% CI 1.19–1.76, *p* < 0.001). The authors identified an optimal cut-off value of 7.7 for MUC in predicting the risk of colectomy with an AUC of 83%, performing better than EMS (71%, *p* = 0.02) [40]. These results strengthen the feasibility of IUS tight monitoring in UC follow-up. This study especially highlighted for the first time the concept that the transmural severity of UC is effectively linked to colectomy risk, pushing forward the relevance of “transmural healing” in UC as settled by the STRIDE II consensus [3]. 

### 3.2. Treatment Response

The final goal enabling the speeding up of decision making in therapeutic adjustment is to be able to perform cross-sectional imaging evaluation of response to treatment in UC, as already available in CD. Several studies have focused their attention on monitoring response to treatment in UC patients, trying to emulate the central role of IUS in CD treat-to-target strategy (Table 2) [41]. The TRUST initiative (transabdominal ultrasonography of the bowel in subjects with IBD to monitor disease activity) was developed to analyze the application of IUS as a non-invasive first-line technique in the tight monitoring and evaluation of treatment response in IBD, both CD and UC [4]. The TRUST study in CD found all ultrasound parameters, including BWT, CDS, BWS and i-fat as correlated significantly at 3 and 12 months with clinical and endoscopic improvement [4]. Moreover, a significant correlation of BWT with serological markers (mainly C-reactive protein [CRP]) was detected [4]. The TRUST-UC study was the first and largest multicenter study, enrolling 253 patients with clinically active UC focusing on IUS as a tool to evaluate treatment response. The authors registered that an improved BWT (<4 mm) in the sigmoid or descending colon resulted correlated with higher rates of clinical response compared to those patients without an improvement of BWT (90.5% vs. 68.9% and 96.4% vs. 68.8%; *p* < 0.001 each) [42]. Moreover, responders showed a reduction in quantitative BWT both for sigmoid or descending colon at week 12 compared to non-responders [42]. Additionally, 84% of patients showing persistent BWT increase at week 12 registered Fecal Calprotectin (FC) levels above 250 µg/g in contrast with only 16.4% of patients with a normalized BWT [42]. The implications of this large, well-designed trial have been game changing in IUS treatment response evaluation of UC. Since the value that the MUC activity score had shown in previous trials in detecting disease activity and predicting long-term outcomes of the disease, its application was analyzed in a cohort of 49 UC patients by Allocca and colleagues [43]. The authors found that MUC < 6.2 was the only independent predictor of EMS ≤ 1 and EMS = 0 at endoscopic reassessment after various biological therapies initiation (OR 5.80, *p* = 0.010 and OR 10.41, *p* = 0.041, respectively) [43]. MUC ≤ 6.2 showed a sensitivity of 67%, a specificity of 78% and an accuracy of 73% in detecting endoscopic improvement at reassessment [43]. Another study by De Voogd et al. was the first to evaluate IUS in assessing treatment response in UC patients under Tofacitinib [44], a JAK inhibitor recently approved for UC [45]. In this paper, the authors were able to identify cut-off values optimal to predict endoscopic remission (EMS = 0), endoscopic improvement (EMS < 2) and endoscopic response (EMS > 1 point of increase) after 8 weeks of induction therapy (BWT = 2.8 mm, AUC 87%, *p* = 0.006; BWT = 3.9 mm, AUC, 92%, *p* < 0.000132; BWT decrease 32%, AUC 87%, *p* = 0.002, respectively) [44]. One of the most delicate settings in UC treatment is the acute severe colitis occurrence with the risk of toxic megacolon leading to urgent surgery to avoid complications [46]. The ability to predict this fearful occurrence embodies great clinical importance. The current European and American guidelines suggest hospitalization and intravenous corticosteroid (SCS) therapy as the optimal management of these acute cases [47,48]. A pilot study on this delicate setting was presented by Scarallo and colleagues, enrolling 52 UC pediatric patients with 69 acute severe colitis events in a 10-year span, treated with various intravenous methylprednisolone regimens [49]. The authors found that IUS features were able to predict corticosteroid therapy failure with great accuracy with respect to clinical assessment, particularly the loss of BWS resulting in the most accurate predictor with specificity and positive predictive value of 97% and 94.4%, respectively [49]. Illvemark and colleagues interestingly explored the efficacy of early intestinal ultrasound evaluation in predicting response to acute colitis intravenous SCS therapy and its role in surgery sparing in adult patients [50]. BWT after 48 ± 24 h after intravenous SCS administration accurately predicted both clinical non-response (pMayo reduction ≤ 30%) and the need for rescue therapy (with Infliximab or Cyclosporine)/urgent surgery (AUC 85%; 95% CI 76%, 95% and AUC 77%, 95% CI 71%, 74%, respectively). The optimal cut-off of BWT identified was >4 mm before starting SCS therapy [50]. These results put on the spotlight a possible role of IUS even in acute UC onset. Another delicate setting to be explored is UC in pregnancy where studies are lacking. With IUS being a non-invasive radiation-free technique, its feasibility in IBD activity assessment and treatment monitoring has been explored [51]. A pilot case report by Gottlieb et al. described the case of a pregnant woman affected by ulcerative pancolitis presenting with an acute flare at week 14 of pregnancy: initial rescue therapy with Infliximab and prednisolone was carried out with early response but subsequent relapse at steroid tapering. Therefore, a dose increment of Infliximab was administered with complete remission of the disease. IUS was performed at every time point of treatment adjustment well describing disease activity and improving patient outcomes [52]. A recent study by Flanagan and colleagues analyzed 71 pregnant IBD patients, of which 24 UC, reporting high accuracy of IUS across the various terms of pregnancy course, with an overall sensitivity of 74% and specificity of 83%, if compared with FC [53]. The most accurate evaluation of treatment response during pregnancy was attempted by De Voogd and colleagues [54]. The authors assessed disease activity during treatment with point-of-care IUS in 39 IBD patients (16 UC), finding a consistent correlation between ultrasound parameters and clinical activity (*r* = 0.60, *p* < 0.0001), with 84% sensitivity and 98% specificity [54]. Crucially, IUS seemed to lose performance during the third trimester compared to the first two. Nevertheless, IUS has demonstrated to be fairly good in describing UC activity during pregnancy with the ability to implement a treat-to-target strategy even in a setting where endoscopy is not viable.

### 3.3. The Role of Histology: Correlation with Transmural Activity?

A heat spot in UC understanding that has recently gathered some attention with respect to CD is the role of histological activity. An important metanalytic effort has explored the controversial role of histology in UC, reporting how almost 30% of UC patients achieving mucosa healing were found as histologically active [55]. From initial reports, the presence of various inflammatory alterations such as decreased levels of mucin, cryptic abscesses and epithelium disruption seemed to be related to long-term relapse of the disease [56], while a recent work proved that basal plasmocytosis was the only relevant relapse predictor [57]. These results suggest that specific histological alterations could be linked to disease course over time. Thus, in the STRIDE II consensus histological healing has been officially ranked among the selected targets to be reached in UC management [3]. Therefore, some efforts have been put in place to assess the bond between transmural evaluation and histologic activity in UC. De Voogd et al. in their groundbreaking paper, notably reported a correlation between BWT and the Robarts Histological Index (RHI) [58] (ρ = 0.49; *p* = 0.002) [44]. Although this outcome is of notice, larger studies with a special focus on the connection between transmural activity in UC and histology are warranted.

## 4. Post-Operative Setting in UC: Pouch Assessment

UC is a complex disease, which rarely but not seldomly faces acute complications like toxic megacolon, or random dysplasia occurrence which still nowadays represents surgical indications [48]. Medical therapy is mainly advisable to prevent emergent colectomy, with elective surgery burdened only by 0.7% of the mortality rate, compared to 5.3% in the emergency setting [59]. Elective three-stage total procto-colectomy with ileo-pouch–anal anastomosis (IPAA) is the gold standard for UC patients with persisting activity, decreasing general clinical conditions, and presenting with random dysplasia at screening colonoscopy, with the risk of CRC occurrence being very high [48]. Although surgery in UC usually provides permanent remission of the disease, often a pouch relapse is possible, not to mention the relevant morbidity following IPAA creation [60]. Strictures and fistulas are among the most frequent post-IPAA complications even in the short term after surgery, advising an early endoscopic evaluation by pouchoscopy, which is still the gold standard for pouch assessment [61]. Besides surgical complications and post-operative setting, inflammation relapse of the pouch is not infrequent, ascribable mainly to chronic pouchitis (up to 60% of cases in UC) [62], but also Crohn’s-like disease or even functional irritable pouch syndrome (IPS) [63]. Pouchoscopy with biopsy sampling allows us to study pouch landmarks areas with a broad evaluation of the whole mucosal surface, to spot inflammation and grade it according to the validated Pouchitis Disease Activity Index (PDAI) [64]. No clear space is given to non-invasive pouch evaluation, with data lacking and not rigorous, especially for ultrasound examinations. Ardalan and colleagues, in an elegantly designed cross-sectional prospective study, first explored the accuracy of IUS in pouch assessment by examining 42 UC patients with IPAA. The patients underwent both trans-abdominal and trans-perineal ultrasound examinations, either in cross-sectional or longitudinal view, with pouchoscopy as a reference standard for outcomes comparison [65]. Concerning the evaluation of pre-pouch ileum, an accurate view was obtained in 97% of patients with trans-abdominal approach: BWT (*p* < 0.072), CDS (*p* < 0.013) and hyperemia (*p* < 0.046) results significantly correlated with the presence of pre-pouch ileitis, while no additional parameter of inflammation assessed (BWS, submucosal prominence, i-fat and absence of peristalsis) was found to be correlated, apart from lymphadenopathy (*p* < 0.034) [65]. At ROC analysis CDS through Limberg score was the most accurate marker of pre-pouch ileitis (AUC 69%). Trans-abdominal IUS also performed above average in discriminating moderate-to-severe inflammation of pre-pouch ileum, identifying a cut-off of 3 mm for BWT to describe severe inflammation with 70% and 84% of sensitivity and specificity [65]. Concerning TPUS, a more accurate evaluation of the body of the pouch and the rectal cuff was reached using a micro-convex probe with respect to the convex probe (85/97% versus 78/25% of patients overall, respectively, *p* = 0.06) [65]. Interestingly only BWT assessed with the convex probe resulted higher in pouchitis versus non-pouchitis group (*p* < 0.001), with fair accuracy for detection of endoscopic and histological signs of acute pouchitis at ROC analysis (AUC 78%, *p* < 0.001). The main cause of inaccurate pouch body and rectal cuff evaluation with the convex probe was due to Body Mass Index (BMI) > 25 kg/m^2^ [65]. The authors were able to assess an increased value of IUS in addition to FC determination in correctly predicting endoscopic and histological active pouchitis, ameliorating sensitivity to 93% and negative predictive value to 83% [65]. This study could potentially pave the way to highlight the interest of IUS and TPUS in pouch assessment in post-surgical UC, also implementing the concept of transmural healing in this setting. 

## 5. Discussion

**Figure 4:**
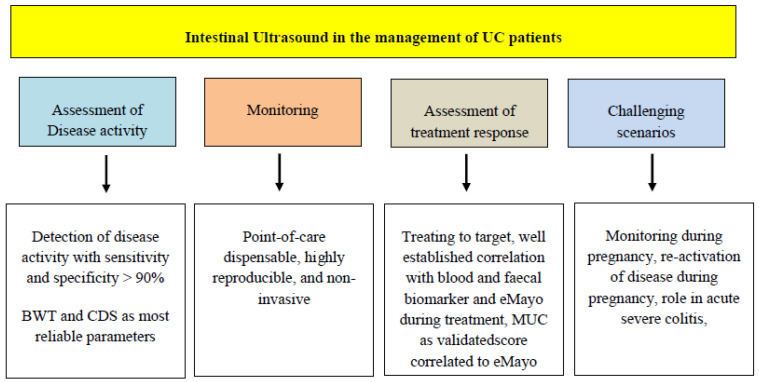
Algorithm on the integration of intestinal ultrasound (IUS) in the management of ulcerative colitis (UC). BWT: bowel wall thickness, CDS: Colour Doppler Signal, eMayo: Mayo endoscopic sub-score.

IUS is a feasible, free-access, easy-to-use tool for the non-invasive assessment of IBD, especially established as a technique of choice in CD, a disease where transmural involvement requires going beyond mucosal healing [66]. Since the non-transmural nature of UC, the positioning of IUS in the diagnostic and therapeutic management of UC patients has been put aside for years. Despite that, increasing interest recently has gathered towards the non-invasive evaluation of UC patients, mostly due to the progressive course of the disease and the increased risk of CRC [67]. The need for fast and efficient decision making for therapeutic adjustments, following specific targets in order to improve long-term outcomes has been confirmed as pivotal in the management of UC as in CD [3]. Colonoscopy still represents the gold standard, but it is unable to provide fastening of the clinical process due to its invasiveness and higher costs. The most recent guidelines advise the implementation of IUS in the work-up of UC as a part of a “treat-to-target” strategy (Figure 4), but are unable to provide a standardization of cut-offs and timing of the examination, due to the paucity of data [9]. Several studies collected evidence on IUS’s role in UC disease activity assessment, using the same parameters assessed for CD, mainly BWT and CDS. A recent comprehensive review of the literature systematically summarizes all the initial experiences of IUS in UC cohorts [16], reporting at least 21 studies, mostly retrospective single-center experiences, in which BWT was described as the main marker correlated with active intestinal inflammation and in 15 of those a broad cut-off of BWT (>4 or >3 mm) was identified as an indicator of active disease with high sensitivity and specificity (up to 89 and 98%) [16]. The need for large cohort, prospective studies evaluating the accuracy of IUS parameters in disease activity assessment and tight monitoring of UC has been accomplished with the two largest and most rigorous experiences by Maaser and colleagues and De Voogd and colleagues, both clearly stating a strong correlation between BWT, empowered by CDS, BWS and i-fat and the response to various treatment regimens in UC patients prospectively [42,44]. These data confirmed previous prospective studies on smaller cohorts but with rigorous design, which reported a predictive value of BWT more than other IUS parameters of endoscopic activity used as reference standard [19,20]. Interestingly, De Voogd and colleagues reported a correlation between treatment response and the reduction of the thickness of the sole submucosal layer of the intestinal wall, paving the way to the upheaval of the concept of UC as merely a mucosal disease, strengthening the importance of its transmural involvement [44]. A blind spot in UC treat-to-target management is still represented by the role of histology. De Voogd et al. reported the notable correlation between IUS parameters and histologic scoring, building the evidence to explore the connection between transmural and histological healing.

Another issue for IUS is the lack of standardization of parameters to meet consensus among IBD ultrasound experts on how to define remission and treatment response. MUC score is the most efficient activity grading score designed specifically for UC able to accurately predict not only disease activity [39] and response to treatment [38], but also the long-term outcome of the disease, especially the risk of colectomy, drastically improving tight monitoring feasibility of the technique [40]. In fact, the capability of safely and non-invasively predicting UC’s long-standing course could potentially reduce hospitalizations, increasing surgical sparing and reducing the need for drastic rescue therapies. In this setting, the results of the studies by Piazza O. Sed and colleagues and Ilvemark and colleagues represented an innovative breakthrough in the understanding of IUS in UC [40,50]. The rectum is the main downside burdening IUS’s role in UC management. The rectum, even if it represents the most frequent localization of UC, several studies have demonstrated it to be less accurately evaluated by ultrasound imaging. TPUS could potentially be the technique of choice to improve the diagnostic ability of ultrasound in rectum assessment, and the pilot data is reassuring [32]. Another burning spot in IUS applied at UC is the absence of a valid methodology to assess intestinal fibrotic modifications that may lead to bowel strictures long term. In CD several studies have explored the role of advanced ultrasound techniques like contrast-enhanced bowel ultrasound (CEUS) [68] or Shear-wave Elastography (SWE) [69] in fibrosis assessment, but data on UC are lacking, limiting the knowledge on the transmural course of this disease. Eventually, post-surgical monitoring by IUS in the clinical setting of pouchitis assessment, to spot relapse distinguish between the real activity of the disease, chronic pouchitis and functional disorders of the pouch. Further efforts have to be made in order to widen the knowledge of IUS use in post-surgical settings in UC. One of the great deals in IUS is the learning curve and the fostering of new ultrasound specialists. IBUS (www.ibus-group.org) is a non-profit organization that for the first time developed a learning curriculum with several standardized targets to be achieved in order to perform bowel ultrasound in autonomy. This organization effort also opened the road to several attempts at learning standardization in other countries, like in Australia with the GENIUS program (https://genius.health).

## 6. Conclusions

IUS is a feasible, accessible, point-of-care tool in UC as in CD. Its role in UC diagnostic and monitoring work-up is currently under standardization. Several studies have extensively demonstrated the accuracy of the several IUS markers available in detecting disease activity and in predicting long-term disease course, in terms of surgery sparing and CRC prevention. The development of accurate activity scores, like the MUC score, is a non-optional improvement for clinical decision making and to standardize therapeutic goals. The outstanding outcome of the largest prospective trials on disease monitoring and therapeutic response assessment encourages the crystallization of this technique for fast assessment and therapy adjustments in the treat-to-target strategy, suggesting IBD specialists move away from the concept of UC as a mere mucosal disease. Further advancements must be acquired to improve IUS accuracy and use with respect to rectum assessment and post-surgical management.

## Figures and Tables

**Table 2:** Retrospective and prospective studies on IUS in treatment response assessment.

Study	Study Type	Cohort N.	Clinical Context	Definition	Type of Treatment	Primary Outcome	Major Findings	Reference
TRUST&UC study-Maaser et al. (Gut 2020)	Multicenter, prospective	253	Active UC	SCCAI ≥ 5	SCS, AZA/6MP, anti-integrin, anti-TNF	% of pts with normalization of BWT in clinical responders (↓ SCCAI by ≥3 points)	✓ 88.5% increased BWT at T0. ✓ Reduction of abnormal BWT after 2 wks: sigmoid colon 89% to 39%; descending colon 83% to 43%; both *p* < 0.001. ✓ Normalization of BWT and clinical response after 12 weeks were highly correlated (*p* < 0.001).	[42]
De Voogd et al. (Gastro 2022)	Multicenter prospective	30	Active UC	EMS ≥ 2 in at least 1 segment	TOF	BWT in SC endoscopic responders versus non-responders at 8 wks	✓ BWT in SC and DC showed moderate to strong correlation with EMS per segment (SC: ρ = 0.68, *p* < 0.0001; DC: ρ = 0.76, *p* < 0.0001). ✓ Median BWT in the SC after 8 wks in ↓EMS vs. ≈ EMS (1.8 mm vs. 4.5 mm, *p* < 0.0001).	[44]
IIlvemark et al. (JCC 2022)	Multicenter prospective	56	Active severe UC	Full Mayo score ≥ 8	SCS	Outcome I: ↓ pMayo score ≥ 30% and ≥3 points, (rectal bleeding subscore of 1 or 0, or a decrease in rectal bleeding subscore ≥1 point) Outcome II: % pts avoiding RT or surgery	✓ Outcome I: after 48 ± 24 h median BWT 3.1 mm vs. 4.9 mm (*p* < 0.0001), haustration (*p* < 0.01), normal BWS (*p* = 0.01), absence of i-fat (*p* = 0.003), in R vs. NR. BWT predicts NR [AUC 0.85 (95% CI 0.76, 0.95)]. ✓ Outcome II: median BWT 3.1 mm vs. 4.4 mm (*p* = 0.002), with CDS = 0 and haustration (*p* < 0.05) in R vs. NR. BWT predicts NR [AUC 0.77, (95% CI 0.71, 0.74)].	[50]
Allocca et al. (JCC 2023)	Single center, prospective	49	Active UC	EMS > 1	IFX, VDZ, ADA, UST	MUC ≤ 6.2 at week 12 predicting EMS ≤ 1 at reassessment	✓ MUC at T0 and w12 8.7, 95% CI 7.7–9.5 vs. 7.5, 95% CI 4.7–8.5 and at reassessment 7.6, 95% CI 4.2–8.9 (*p* < 0.001). ✓ MUC ≤ 6.2 predictor of EMS < 1 at w12 (OR 5.80, 95% CI 1.49–22.47 (*p* = 0.01) and EMS = 0 (OR 10.41, 95% CI 1.09–99.29 (*p* = 0.041).	[43]

ADA: Adalimumab; AZA: Azathioprine; AUC: Area Under Curve; BWS: Bowel Wall Stratification; BWT: Bowel Wall Thickness; CI: Confidence Intervals; DC: Descending Colon; EMS: Endoscopic Mayo Score; IFX: Infliximab; MUC: Milan Ultrasound Criteria; NR: Non-responders; OR: Odds Ratio; PTS: Points; R: Responders; RT: Rescue Therapy; SCCAI: Short Clinical Colitis Activity Index; SC: Sigmoid Colon; SCS: Systemic Corticosteroids; TNF: Tumour Necrosis Factor; TOF: Tofacitinib; T0: Baseline; UC: Ulcerative Colitis; UST: Ustekinumab; VDZ: Vedolizumab; 6MP: 6-Mercaptopurine.

## Data Availability

No new data were generated or analyzed in support of this research.
